# Molecular ageing in progeroid syndromes: Hutchinson-Gilford progeria syndrome as a model

**DOI:** 10.1186/1742-4933-6-4

**Published:** 2009-04-20

**Authors:** Henrique Douglas M Coutinho, Vivyanne S Falcão-Silva, Gregório Fernandes Gonçalves, Raphael Batista da Nóbrega

**Affiliations:** 1 Laboratório de Pesquisa em Produtos Naturais, Departamento de Ciências físicas e Biológicas, Centro de Ciências Biológicas e da Saúde, Universidade Regional do Cariri, Crato (CE), Brazil; 2 Laboratório de Genética de Microrganismos, Departamento de Biologia Molecular, Centro de Ciências Exatas e da Natureza, Universidade Federal da Paraíba, João Pessoa (PB), Brazil; 3 Laboratório de Bioquímica, Genética e Radiobiologia, Departamento de Biologia Molecular, Centro de Ciências Exatas e da Natureza, Universidade Federal da Paraíba, João Pessoa (PB), Brazil

## Abstract

Hutchinson-Gilford progeria syndrome (HGPS) is a rare premature aging disorder that belongs to a group of conditions called laminopathies which affect nuclear lamins. Mutations in two genes, LMNA and ZMPSTE24, have been found in patients with HGPS. The p.G608G LMNA mutation is the most commonly reported mutation. The aim of this work was to compile a comprehensive literature review of the clinical features and genetic mutations and mechanisms of this syndrome as a contribution to health care workers. This review shows the necessity of a more detailed clinical identification of Hutchinson-Gilford progeria syndrome and the need for more studies on the pharmacologic and pharmacogenomic approach to this syndrome.

## Introduction

Biological ageing considers that some ageing-associated changes are programmed while others are stochastic and unpredictable [1,2]. An alternative to the analysis of aging is the study of human genetic syndromes whose phenotypes show specific characteristics of human ageing. Premature ageing syndromes (or progeroid syndromes) constitute one of these alternatives since they are considered to be segmental progeroid syndromes [3].

## Hutchinson-Gilford progeria syndrome (HGPS)

Hutchinson-Gilford progeria syndrome (HGPS; OMIM #176670) is a very rare genetic disease, characterized by precocious ageing in early infancy, with several clinical characteristics such as growth retardation after birth, failure to thrive and skin abnormalities (sclerotic skin dimpling and mottling), sleeping with eyes open, circumoral cyanosis, prominent eyes and cutaneous/scalp vasculature, decreased joint range of motion, micrognathia, premature atherosclerosis, loss of subcutaneous fat, alopecia, fingertip tufting, distal-joint abnormalities, altered pigmentation and generalized anomaly in bone development with pathogenic fracture and osteolysis [4-7].

The syndrome name is derived from the Greek word *geras*, meaning "prematurely old." It was first described by Jonathan Hutchinson in 1886, and named by Hastings Gilford in 1904. Since its first description in 1886, over 150 cases have been reported in the world [5,8]. These patients have a short life expectancy with death occurring in adolescence, most times due to cardiovascular diseases [7].

There are other forms of progeria that manifest at several ages and are characterized by signs of ageing, such as the following syndromes: Wiedemann-Rautenstrauch, Cockayne, Werner, Emery-Dreifuss, Rothmund-Thomson, and Seckel [5].

The incidence of classic progeria has been estimated to be 1 per 4–8 million live births [9]. Prevalence of sex has not been evidenced so far. The majority of HGPS patients are Caucasian [4,5,10]. Over 150 cases have been reported in the literature throughout the world [11]. The average life expectancy for a patient with HGPS is 13 years, with an age range of 7 to 27 years old. Caucasians represent 97% of patients with HGPS, and cardiovascular abnormalities account for 75% of death in patients with the syndrome [10].

The objective of this present work was to carry out a literature review of the molecular traits and clinical features of Hutchinson-Gilford progeria syndrome (HGPS), in a search based on mutations in the genes involved with this genetic disease.

## Methodology

The keywords used for this review were progeria, ageing, LMNA gene and Hutchinson-Gilford. The search was performed using the international bibliographic databases and was started in the 1990s and updated to 2008.

## Molecular mechanism of laminopathies

**Figure 1:**
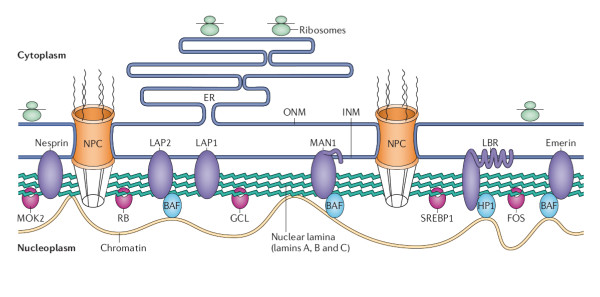
**Structure and function of the nuclear lamina**. The nuclear lamina lies on the inner surface of the inner nuclear membrane (INM), where it serves to maintain nuclear stability, organize chromatin and bind nuclear pore complexes (NPCs) and a steadily growing list of nuclear envelope proteins (purple) and transcription factors (pink). Nuclear envelope proteins that are bound to the lamina include nesprin, emerin, lamina-associated proteins 1 and 2 (LAP1 and LAP2), the lamin B receptor (LBR) and MAN1. Transcription factors that bind to the lamina include the retinoblastoma transcriptional regulator (RB), germ cell-less (GCL), sterol response element binding protein (SREBP1), FOS and MOK2. Barrier to autointegration factor (BAF) is a chromatin-associated protein that also binds to the nuclear lamina and several of the aforementioned nuclear envelope proteins. Heterochromatin protein 1 (HP1) binds both chromatin and the LBR. ONM, outer nuclear membrane [9].

HGPS belongs to a group of disorders called laminopathies which affect nuclear lamins and include several phenotypes (figure 1). Mutations in the gene *LMNA *have been identified in the majority of cases of HGPS. The gene *LMNA *encodes nuclear lamin A, with the predominant somatic cell isoforms lamin A and C arising by alternative RNA splicing, which underlies and organizes the inner surface of the nuclear envelope [12-14]. There are at least 11 distinct diseases associated with >300 different mutations in *LMNA *[15-17]. Mutations in *LMNA *have been detected in 88% of patients with HGPS, while the genetic mechanism of the remaining 12% is still unknown [3].

**Figure 2:**
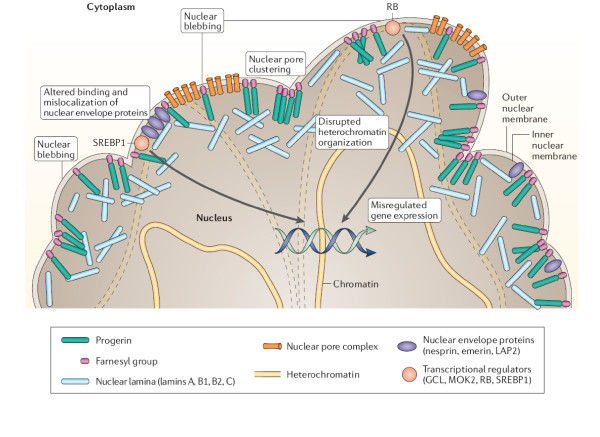
**Potential mechanisms of disease**. A range of molecular and cellular mechanisms are likely to contribute to the diverse phenotypes that are seen in the laminopathies, and these mechanisms probably vary depending on the specific mutation. Here, as an example, we show a range of putative disease-causing mechanisms for the case of HGPS, in which lamin A is permanently farnesylated in the form of progerin. We predict that progerin becomes entrapped in the nuclear membrane as a result of permanent farnesylation, resulting in a multitude of downstream effects. Disruption of the normal lamina architecture leads to fragility, vulnerability to mechanical stresses and nuclear blebbing. Other consequences include disrupted interactions with other nuclear envelope proteins – such as nesprin, emerin and laminaassociated protein 2 (LAP2) – which leads to their mislocalization (that is, emerin is relocalized to the cytoplasm in Lmna -/- mice)74 and clustering of nuclear pores. Disorganization and loss of peripheral heterochromatin is also seen, with heterochromatin becoming detached from the nuclear envelope, and disrupted interactions with RNA polymerase II, RNA splicing factors and transcription factors such as the retinoblastoma transcriptional regulator (RB) and sterol response element binding protein (SREBP1), which leads to misregulation of gene expression. GCL, germ cell-less [9].

Lamins are structural components of the nuclear lamina, a proteinaceous network underlying the inner nuclear membrane which determines the shape, integrity and size of the nucleus. Furthermore, lamin plays an important role in the organization of the pore complex, and recruits other proteins such as emerin for the nuclear envelope (figure 2). Lamins A and C are important for nuclear stability, play a role in transcription regulation in response to a chemical or mechanical stimulus, and are members of the intermediate-type filament proteins which also form part of the nuclear matrix scaffold [15,16]. Due the multiple interactions between chromatin and the nuclear matrix, mutations in lamins A and C are thought to impair various nuclear functions including chromatin and chromosome stability, telomere integrity, regulation of transcription, DNA replication, cell cycle control and cellular differentiation, causing a variety of disorders which first affect striated muscle, adipocytes and peripheral nerves or cause the appearance of premature aging diseases called laminopathies. A single mutation in lamin A is the cause of HGPS [3,18,19].

Lamin A in mammals is an element of the polypeptide family of lamins. Its main components are lamins A, B1, B2 and C, with molecular weight ranging from 60,000 to 78,000. Lamins A and C are formed by the splicing of lamin A mRNA. Lamins B1 and B2 are coded by separate genes, while lamins A and C are identical in the first 566 amino acids. Lamin A is normally synthesized as a precursor molecule (prelamin A). Alternative splicing in exon 11 causes an increase in two different mRNAs which code for prelamin A and lamin C. Prelamin A, with 664 amino acids, has 98 carboxyl-terminal amino acids, while lamin C has 6 carboxyl-terminal amino acids. As lamin A contains a carboxyl-terminal CAAX box (C is cysteine, A is an aliphatic amino acid and X is any amino acid), it is modified by farnesylation, which does not occur in lamin C. Following farnesylation, the cleavage of the three last amino acids, and methylation of the carboxyl-terminal, an internal proteolytic cleavage takes place removing the last 15 coding amino acids, in order to generate a mature lamin A with 646 amino. Progerin, the altered product in HGPS, is an incompletely processed lamin A/C that remains farnesylated, leading to apparent loss of mechanical properties of the nuclear envelope and nuclear matrix. Cells expressing progerin may experience delayed mitotic progression which would be consistent with the early onset and global growth deficit of the HGPS phenotype [20].

The mutation G608G of HGPS and the consequent abnormal splicing produce a prelamin A that still retains the CAAX box, but is missing a part for endoproteolytic cleavage. Immunofluorescence of HGPS fibroblasts with lamin A antibodies revealed that 40–50% of the cells displayed a visible abnormality, an abnormal nuclear envelope morphology. Thus, this mutation in HGPS seems to act as a dominant-negative mutation that affects nuclear morphology [21].

Affected nuclear mechanisms and secondary alterations in gene expression may cause damage to the striated muscle of individuals with certain mutations in lamin A/C. Different mutations in lamins A and C may cause various tissue-specific pathological phenotypes [19]. There are several reports about the nuclear abnormalities such as lobulation or "blebbing" of the nuclear envelope, increased nuclear surface area, thickening of the nuclear lamina, loss of peripheral heterochromatin and clustering of nuclear pore complexes [6,22].

**Figure 3:**
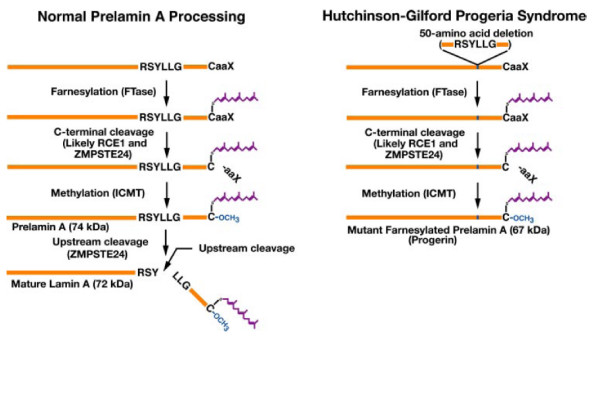
**Biogenesis of lamin A in normal cells and the failure to generate mature lamin A in HGPS**. In the setting of ZMPSTE24 deficiency, the final step of lamin processing does not occur, resulting in an accumulation of farnesyl-prelamin A. In HGPS, a 50-amino acid deletion in prelamin A (amino acids 607–656) removes the site for the second endoproteolytic cleavage. Consequently, no mature lamin A is formed, and a farnesylated mutant prelamin A (progerin) accumulates in cells [25].

HGPS is related to a mutation in the *LMNA *gene or in ZMPSTE2 that codes for a metalloproteinase specifically involved in the post-translational proteolytic processing of prelamin A to mature lamin A, which is responsible for scaffolding and organizing the nuclear envelope surface (figure 3) [12,23-25].

The inheritance pattern in progeria syndrome is autosomal dominant (or less frequently recessive when involving the ZMPSTE24 gene) [22,26]. All subjects with HGPS have the disease as result of a *de novo *mutation (the most common mutation is p.G608G), as their parents are not affected. This mutation causes aberrant splicing in exon 11 and the deletion of 50 residues close to the C terminus of lamin A, including the second ZMPSTE24 cleavage site. This deletion prevents complete processing of prelamin A, resulting in the accumulation of a farnesylated lamin A, known as progerin. Five other different de novo dominant LMNA mutations have been found less frequently: p.E145K, p.S143F, p.R644C, p. T10I and p.E578V [13]. Despite being very rare, mutations in HGPS are thought to have a paternal origin [27].

## Clinical manifestations

Patients with HGPS are infants who are healthy at birth and in the course of 1–2 years present signs of progressive premature ageing. Initially, sclerodermatous plaques appear on the skin of the hip and in the upper region of the lower extremities. These areas grow more and put virtually the entire body at risk, except for the genitals and some regions of the lower limbs. The production of sweat is simultaneously decreased and alopecia becomes evident. Some late signs are hyperpigmentation in sun-exposed areas, as well as dystrophic nails [5]. The clinical manifestations of classic progeria include abnormalities in growth, skin and skeletal and cardiovascular systems, which are always present after the age of 3 years [6].

The ever present clinical features are prominent scalp veins, alopecia, bird-like facies, prominent eyes, abnormal dentition and delayed tooth eruption, micrognathia, short clavicles, horseman stance, pyriform thorax, thin legs with prominent joints, short stature and low weight for height, incomplete sexual maturation and lack of subcutaneous fat. The clinical manifestations that may be apparent or not are sclerodermatous skin, generalized alopecia, eyelashes and eyebrow alopecia, protruding ears with absent lobes, beaked nose, thin lips with centrofacial cyanosis, protracted anterior fontanel and high-pitched voice [4,7,10].

Patients who have most of the aforementioned characteristics are considered to have a classic case of progeria. However, individuals who have characteristics more or less intense of the syndrome are considered patients with atypical progeria [21].

## Diagnostic methods

The diagnostic methods of HGPS are clinical (serum lipid levels, hyaluronic acid excretion, blood count), histological (biopsies from areas of abdominal skin with abnormal nuclear morphology), radiological (abnormality found in the brain, thorax, long bones and phalanges) and by screening for mutations in the gene *LMNA *[12-14].

No laboratory offers a specific molecular genetic testing for prenatal diagnosis of progeria. Nevertheless, prenatal testing may be offered to families in which the causative mutation of the disorder has been identified in a family member [13].

Some clinical tests for confirmatory diagnosis are sequential analysis of the gene *LMNA*, which reveals point mutations in approximately 90% of the patients with HGPS, and the test for uniparental disomy of chromosome 1 and deletions associated with HGPS. Imaging studies may also be performed. Radiography detects manifestations that usually occur in the skull, thorax, long bones, and phalanges [10].

Under light microscopy, histological tests using skin biopsies from HGPS patients exhibit irregular nuclear envelope outlines, indicating the massive and global alterations of chromatin functions, including alterations of gene expression [20]. Tests using keratinocytes from transgenic mice expressing progerin revealed alterations in nuclear shape such as decreased nuclear circularity, resulting in greater nuclear surface area and greater morphological diversity, thereby, microscopic analysis of the nuclear shape could be an interesting diagnostic alternative to be studied [14].

## Treatment

There is no known cure for progeria. Nonetheless, there are treatments in order to improve the clinical conditions. Regular diets may be prescribed, as well as routine immunizations, inspection for cardiovascular diseases, treatment with aspirin, surgical procedures, and physical and psychological therapies. Children with HGPS must have a regular diet. Common mulltivitamin tablets are appropriately given in normal doses. Supplements with fluoride are recommended, since there are dental problems. It is advised to occasionally give small doses of aspirin to children with HGPS, aimed at reducing the occurrence of heart attack and strokes. Atherosclerosis of the coronary artery may be diagnosed with an echocardiogram (ECG), and nitroglycerin may be useful should there be development of angina. The drug doses must be based on weight and the anesthetics must be used cautiously. As these children are susceptible to fractures, they should be routinely accompanied. Due to the susceptibility to dislocation of the hip bone because of coxa valga, conservative care and surgical procedures are recommended. In relation to delay or loss of the first dentition, dental extraction may be recommended. Physical and psychological therapies are recommended to help maintain the joints with good movement amplitude, as well as to foster social interaction, respectively [21].

Another therapeutic approach involves the use of farnesyltransferase inhibitors which have been shown to reverse abnormalities in nuclear morphology in cells expressing progerin [14]. *In vitro *studies in fibroblasts have shown the capacity of farnesyltransferase inhibitors (FTI) to reverse nuclear alterations [28-30].

Using transgenic mice expressing progerin, an ammelioration and reversion of cardiovascular phenotype, number reduction of the spontaneious rib fractures and improved survival and growth was observed, indicating that these compounds are an interesting pharmacological alternative for future treatment of HGPS and progeroid syndromes, as well as anti-aging [14,31,32]. The promising results with FTIs led to an open-label clinical trial of the FTI use in HGPS (ClinicalTrials.gov number NCT000425607) [7].

Other possibilities of treatments studied involves the use of low levels of growth hormone, but hormonal replacement has had unsatisfactory effects in these patients [10,11]. The use RNAi or antisense strategies might be useful for reduce the progerin production in HGPS patients, but this strategy have few studies [31,33,34]. The combined use of a statin and a biphosphonate, compounds that act in the same mevalonate pathway as FTIs, but the effect of these drugs alone and combined to treat HGPS remains an open question [32].

## Prognostic

The causes of morbidity in HGPS are difficulty in development, cerebrovascular events, vertigo, migraine, necrosis of the head of the femur and luxation of the hip [5].

Despite all medical and technological advances in cardiovascular surgeries (catheterization, cardiac pacemaking), improvement in patients' life expectancy has not been achieved, due to their tendency to accumulate atheromatous plaques again. The main cause of death (in 75% cases) is cardiac or cerebrovascular abnormalities which include myocardial infarction and congestive heart failure due to premature atherosclerosis [13]. Psychological support in these patients is important, as well as a good family circle, since there is no mental abnormality. However, they do have deficient body and physiopathologic control [5].

## Final considerations

While there is a move towards precise molecular classification of "laminopathies" rather than the broad clinical categories in use to date, it appears essential that clinical features be documented in detail. This may help in answering parents' questions about prognosis. It may also contribute to a better understanding of the functions of lamin A, and may in the future help in determining the efficacy of pharmacogenetic interventions for Hutchinson-Gilford syndrome and other progeroid syndromes. Due the fact of the cencentration of progering is enhanced in fibroblast of older persons, HGPS and possibly other laminopathies may serve as a model for the normal ageing process.

## Competing interests

The authors declare that they have no competing interests.

## Authors' contributions

VSFS, RBN and GFG contributed to conception and design, designed the review, carried out the literature research, and manuscript preparation. HDMC contributed to conception and design, carried out the manuscript editing and manuscript review. All authors read and approved the final manuscript. We declare that all figures used in this article were authorized by the corresponding authors of the cited articles, Dr. Stephen G. Young and Dr. Francis S. Collins.
